# Polycystin-2 Is Required for Starvation- and Rapamycin-Induced Atrophy in Myotubes

**DOI:** 10.3389/fendo.2019.00280

**Published:** 2019-05-08

**Authors:** Catalina Kretschmar, Daniel Peña-Oyarzun, Cecilia Hernando, Nadia Hernández-Moya, Alfredo Molina-Berríos, María Paz Hernández-Cáceres, Sergio Lavandero, Mauricio Budini, Eugenia Morselli, Valentina Parra, Rodrigo Troncoso, Alfredo Criollo

**Affiliations:** ^1^Facultad de Odontología, Instituto de Investigación en Ciencias Odontológicas, Universidad de Chile, Santiago, Chile; ^2^Facultad Ciencias Químicas y Farmacéuticas and Facultad Medicina, Advanced Center for Chronic Diseases, Universidad de Chile, Santiago, Chile; ^3^Departamento de Fisiología, Facultad de Ciencias Biológicas, Pontificia Universidad Católica de Chile, Santiago, Chile; ^4^Facultad de Medicina, Centro de Estudios en Ejercicio, Metabolismo y Cáncer, Universidad de Chile, Santiago, Chile; ^5^Cardiology Division, Department of Internal Medicine, University of Texas Southwestern Medical Center, Dallas, TX, United States; ^6^Autophagy Research Center, Santiago, Chile; ^7^Laboratorio de Investigación en Nutrición y Actividad Física, Instituto de Nutrición y Tecnología de los Alimentos, Universidad de Chile, Santiago, Chile

**Keywords:** atrophy, polycystin-2, myotubes, mTOR, starvation, rapamycin

## Abstract

Muscle atrophy involves a massive catabolism of intracellular components leading to a significant reduction in cellular and tissue volume. In this regard, autophagy, an intracellular mechanism that degrades proteins and organelles, has been implicated with muscle breakdown. Recently, it has shown that polycystin-2 (PC2), a membrane protein that belongs to the transient receptor potential (TRP) family, is required for the maintenance of cellular proteostasis, by regulating autophagy in several cell types. The role of PC2 in the control of atrophy and autophagy in skeletal muscle remains unknown. Here, we show that PC2 is required for the induction of atrophy in C2C12 myotubes caused by nutrient deprivation or rapamycin exposure. Consistently, overexpression of PC2 induces atrophy in C2C12 myotubes as indicated by decreasing of the myogenic proteins myogenin and caveolin-3. In addition, we show that inhibition of mTORC1, by starvation or rapamycin is inhibited in cells when PC2 is silenced. Importantly, even if PC2 regulates mTORC1, our results show that the regulation of atrophy by PC2 is independent of autophagy. This study provides novel evidence regarding the role of PC2 in skeletal muscle cell atrophy.

## Introduction

Atrophy defined as a decrease in the mass and size of tissues or cells, is caused by a massive loss of proteins, cytoplasm, and organelles. Muscle cells respond to different pathophysiological stimuli by activating pathways involved in protein degradation. Stimuli such as cancer pharmacological treatments, AIDS, sepsis, heart failure, burn injury, and multiple sclerosis among others can induce severe muscle atrophy (1–4). The preservation of the homeostasis in muscle cells is crucial, not only because of the maintenance of an optimal muscle performance, but also because muscle represents an important source of amino acids and nutrients, which can be metabolized by different organs such as brain, heart and liver (5). A severe or aggressive episode of atrophy can aggravate other co-lateral diseases, and seriously increase morbidity and mortality. Importantly, massive macroautophagy, hereafter referred as autophagy, is one of the mechanisms involved in muscle cell atrophy (6). Autophagy is a fundamental intra cellular process for degrading and recycling components such as proteins, organelles, and cytoplasm. Autophagy is characterized by the formation of autophagosomes, which fuse with the lysosome to form the autolysosome where the intravacuolar material is degraded. Studies *in vitro* and *in vivo* have shown that there is a strong relationship between autophagy and skeletal muscle atrophy (7–9). Studies in C2C12 myotubes and a murine cancer model showed that activation of autophagy contributes to muscle wasting in cancer cachexia (10). Consistently, other studies have shown that the mechanistic target of rapamycin complex 1, mTOR, a constitutive kinase protein that inhibits autophagy, blocks atrophy in muscle and other cell types (11). In this regard, muscle-specific mTOR knockout mice present a severe muscle atrophy phenotype. Consistently, the insulin- or IGF-1-induced overactivation of mTOR blunts atrophy through the inhibition of autophagy in cardiac muscle (12, 13). Furthermore, inhibition of mTOR, induced by nutrient restriction or treatment with rapamycin (which inhibits mTORC1), causes autophagy and atrophy in skeletal muscle (11, 12, 14, 15). These evidences indicate the existence of a cross-talk between mTOR, autophagy and atrophy in the control of metabolism and cell and tissue size. Although different signaling pathways have been elucidated during the process of atrophy, the molecular mechanisms by which they can be modulated are still unknown. Recently, we have showed that the protein polycystin-2, PC2, a member of the transient receptor potential family which acts as a non-selective cation channel, is required for nutrient deprivation-induced autophagy *in vivo* and by hypertonicity, rapamycin and starvation *in vitro* in different types of cells (16, 17). Others studies also supported the role of PC2 in the regulation of autophagy. Indeed in renal epithelial cells and human embryonic stem cell-derived cardiomyocytes, PC2 is required for fluid flow- and glucose starvation-induced autophagy, respectively (18, 19).

PC2 regulates autophagy by activating different pathways; among those are listed classic (mTOR-dependent) and mTOR independent pathways, such as Ca^2+^-dependent modulation of autophagy. Interestingly, as PC2 is not an ubiquitous protein and since PC2 can regulate autophagy thanks to its Ca^2+^ channel function, the modulation of autophagy by PC2 can be cell, tissue and stimuli dependent (16–19).

Here, we show that PC2 is required for starvation- and rapamycin- induced atrophy and inactivation of mTOR in C2C12 myotubes, without affecting the modulation of autophagy in the same cell type.

## Results

### Classical Autophagic Inducers Causes Atrophy in C2C12 Myotubes

**Figure 1:**
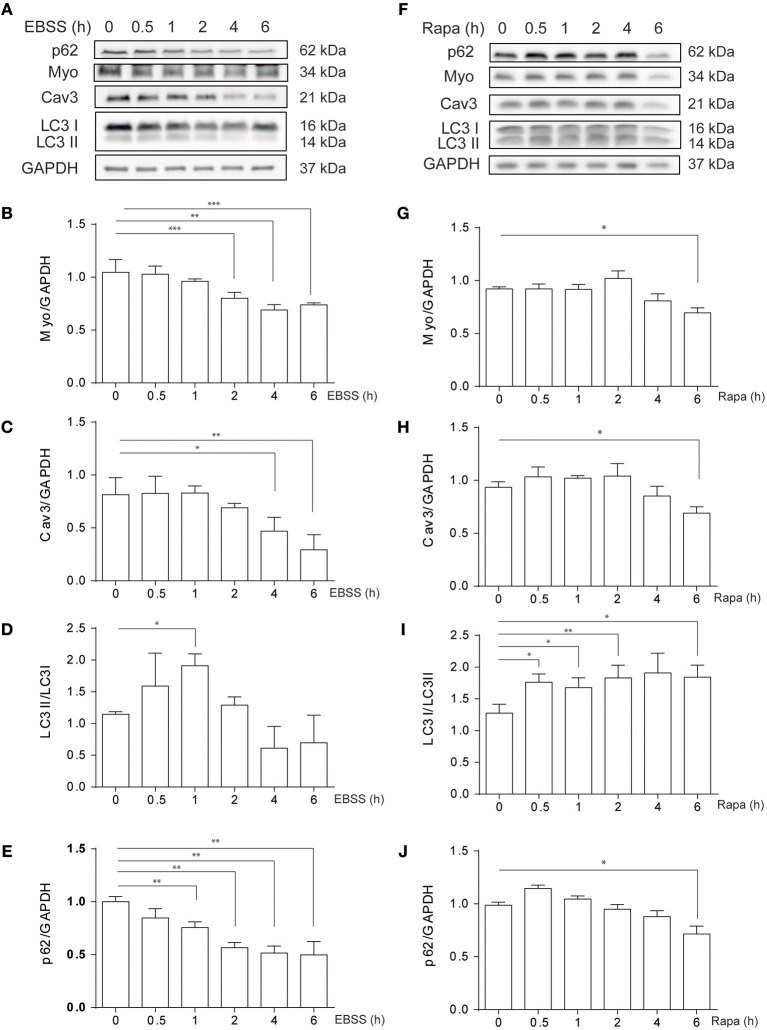
Starvation- and rapamycin-induced autophagy and atrophy in C2C12 myotubes. C2C12 myotube cultures were submitted to starvation with EBSS **(A–E)** or treated with rapamycin 0.1 μM **(F–J)** by 0, 0.5, 1, 2, 4, and 6h. Subsequently, starvation- and rapamycin- induced atrophy were evaluated by western blot, by evaluating the levels of myo **(A,B,F,G)** and Cav3 **(A,C,F,H)**. Autophagy was evaluated by western blot by assessing the levels of LC3 I and II **(A,D,F,I)**. GAPDH was used as loading control. Representative gels are showed in **(A)** and **(F)** and gels quantifications are depicted in **(B–E)** and **(G–J)**, respectively (mean± S.E.M., *n* = 3, *p < 0.05, **p < 0.01, and **p < 0.001).

C2C12 myotubes were exposed to classical autophagy inducers such as nutrient deprivation, by treatment with Earle's Balanced Salt Solution (EBSS) or by pharmacological inhibition of mTORC1 with rapamycin, 0.1 μM, at different time points (0–6h) (Figure 1). Protein levels of myogenic markers Myogenin (MYO) and Caveolin-3 (CAV3), which are down-regulated in different *in vitro* and *in vivo* models of atrophy, decrease in C2C12 myotubes following EBSS or rapamycin exposure (Figures 1A–J). In addition, as expected, both EBSS and rapamycin induce autophagy in C2C12 myotubes as assessed by the analysis of LC3 I to LC3 II conversion by western blotting, reaching the highest level at 0.5 and 1h post-treatments (Figures 1A,D,F,I). We also analyzed the levels of p62/SQSTM1, a protein that binds specifically to LC3 and thus is degraded in the autolysosome (20). Consistently, the level of p62/SQSTM1 decreases when cells are exposed to EBSS or treated with rapamycin (Figures 1A,E,F,J), confirming that autophagy is up-regulated in C2C12 myotubes following the aforementioned treatments. Altogether these data show that nutrient deprivation and rapamicyn not only induce autophagy but also atrophy in C2C12 myotubes.

### PC2 Deficiency Prevents Atrophy in an Autophagy-Independent Manner in C2C12 Myotubes

**Figure 2:**
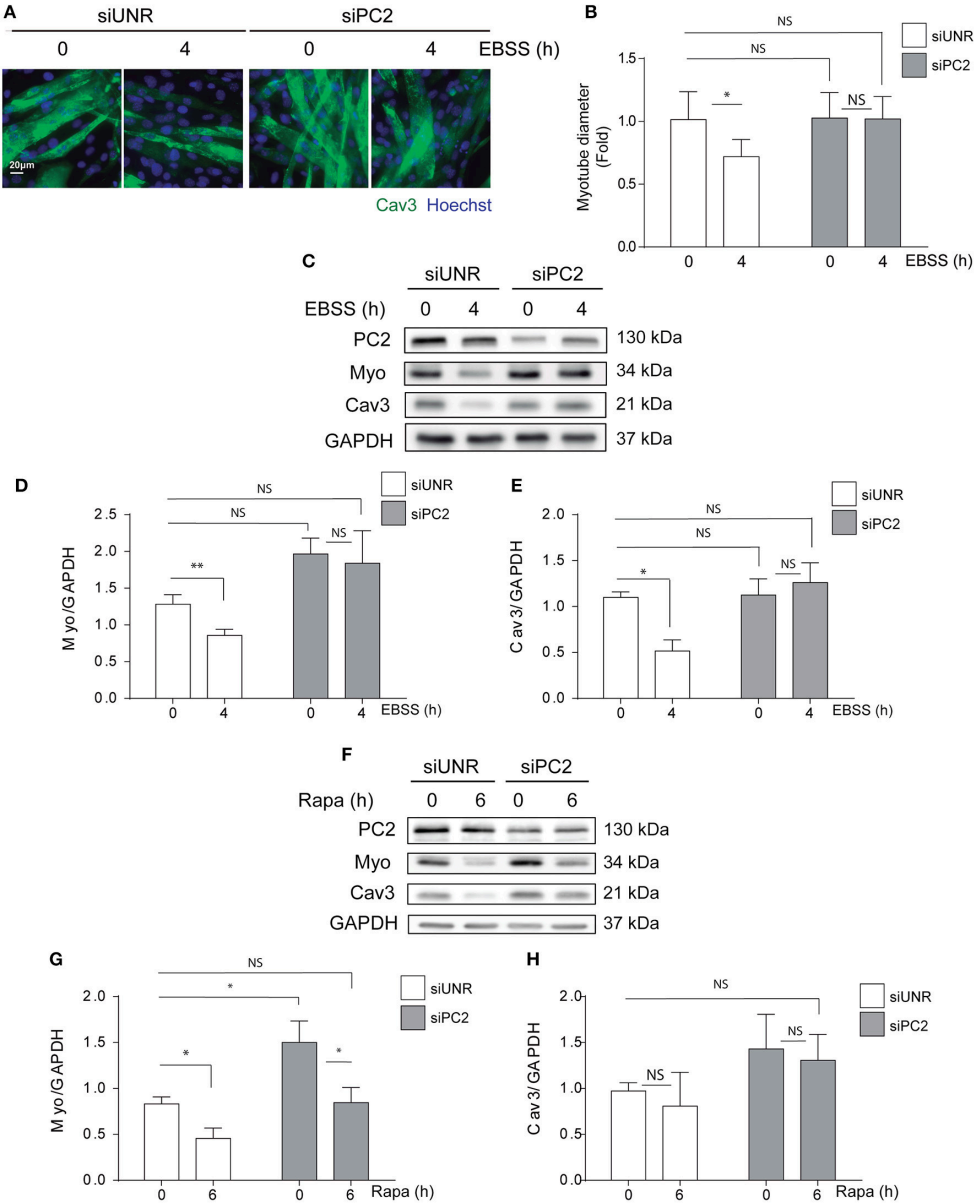
PC2 is required for atrophy but not autophagy in C2C12 myotubes. PC2 was downregulated in C2C12 myotube cultures using a specific siRNA against PC2 (siPC2). An unrelated siRNA (siUNR) was used as control. Subsequently, 48h post-transfection cells were subjected to nutrient deprivation by incubation in EBSS for 0 and 4h **(A–D)** or treated with rapamycin 0.1 μM for 0 and 6h **(F–H)**. **(A,B)** Cells were fixed and immunostaining against caveolin-3 (Cav3) was performed to evaluate myotube diameter by fluorescent microscopy. Nuclei were stained with 1 μg/mL Hoechst 33342. Representative pictures are showed in **(A)** and myotube diameter quantification is represented in **(B)** (mean± S.E.M., *n* = 3, *p < 0.05). **(C,D)** Whole lysates were resolved by western blot and Myogenin (Myo), Cav-3, LC3 I, and LC3 II levels were evaluated by the use of specific antibodies. GAPDH was used as a loading control. Representative gels are showed in **(C,F)** and gels quantifications are depicted in **(E,D,H,G)** (mean± S.E.M., *n* = 3, *p < 0.05, **p < 0.01).

PC2 is a member of the TRP channels protein family and studies have shown that PC2 is involved in mechanisms of mechanotransduction mostly in renal epithelial cells (21, 22). In addition, recently we and others have shown that PC2 regulates autophagy in different cell types (16, 17, 19). Indeed, we showed PC2 is required for hyperosmotic stress-induced autophagy in human cervical and colon cancer cell lines, HeLa and HCT116, respectively (16). Furthermore, it has been shown that PC2 is required for autophagy induction also in primary rat neonatal cardiomyocytes submitted to starvation or exposed to rapamycin (17), as well as in kidney mouse epithelial cells exposed to changes in fluid flow (18). In addition, animals knocked-out for PC2 in cardiomyocytes are resistant to starvation-induced autophagy, specifically in the heart (17). Given that PC2 regulates autophagy, and that autophagy is enhanced by action of different atrophy inducers in skeletal muscle cells, we evaluated if PC2 is required for atrophy in C2C12 myotubes exposed to nutrient deprivation or rapamycin. Our results showed that down regulation of PC2, by the use of specific siRNAs, prevented atrophy as indicated by the myotube diameter in C2C12 cells submitted to starvation (Figures 2A,B). In addition, western blot assays revealed that the decrease in the levels of MYO and CAV3 in cells exposed to nutrient deprivation or rapamycin for 4 and 6h, respectively, is inhibited in C2C12 myotube cultures transfected with a specific siRNA against PC2 (siPC2) (Figures 2C–H). Despite the effects of PC2 in the regulation of atrophy, we did not observe inhibition of starvation- and rapamycin-induced autophagy, evaluated by the conversion of LC3 I to LC3 II, in cells down-regulated for PC2 (Supplementary Figure 1). Altogether these results show that PC2 is required for starvation- and rapamycin-induced atrophy in an autophagy-independent manner in C2C12 myotubes.

### PC2 Overexpression Induces Atrophy in C2C12 Myotubes

**Figure 3:**
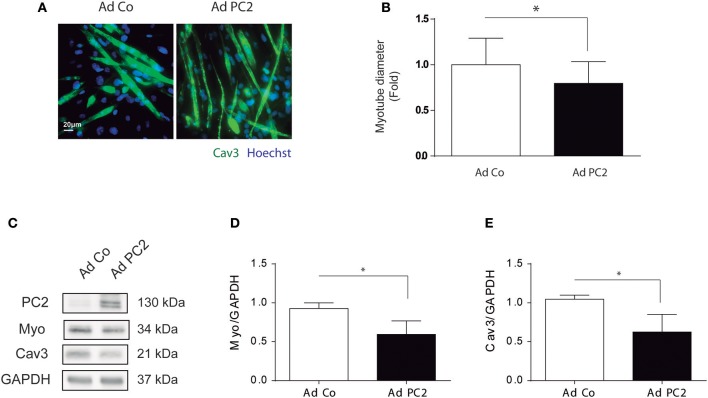
Overexpression of PC2 induces atrophy and autophagy in C2C12 myotubes. PC2 was overexpressed in C2C12 myotubes by adenoviral transduction with Ad PC2 and an empty adenovirus was used as control (Ad Co) **(A–E)**. Twenty-four hours post transduction cells were fixed and immunostained against Caveolin-3 (Cav3), subsequently myotube diameter was evaluated by fluorescent microscopy. Nuclei were stained with 1 μg/mL Hoechst 33342. Representative pictures are showed in **(A)** and myotube diameter quantification is showed in **(B)** (mean ± S.E.M., *n* = 3, *p < 0.05). **(C–E)** Whole lysates were resolved by western blot and Polycystin-2 (PC2), Myogenin (Myo), and Cav3 levels were evaluated by the use of specific antibodies. GAPDH was used as a loading control. Representative gels are showed in **(C)** and gels quantifications are depicted in **(D,E)** (mean ± S.E.M., *n* = 3, *p < 0.05).

Our results showed that PC2 is required for starvation- and rapamycin-induced atrophy in C2C12 myotubes (Figure 2). However, if PC2 overexpression is sufficient to induce atrophy in C2C12 myotubes remains elusive. To this aim, we overexpressed PC2 by the use of the adenovirus, Ad PC2, or control adenovirus, Ad Co, and we evaluated atrophy in C2C12 myotubes. Our data showed that over expression of PC2 induced a reduction in myotubes diameter (Figures 3A,B), which correlated with a decrease in the levels of MYO and CAV3 (Figures 3C–E). Furthermore, PC2 overexpression did not induce autophagy, as evaluated by LC3 I to LC3 II turnover, indicating that regulation of autophagy is not involved as mechanism by which PC2 regulates atrophy in C2C12 myotubes (Supplementary Figures 2A,B). Given that C2C12 myotubes are a syncytium formed by the fusion of multiple cells, it is possible that they might be more resistant to autophagy. Thus, we also evaluated if PC2 induces autophagy in myoblasts. Our results showed that, similar to the results obtained in myotubes, overexpression of PC2 did not induce autophagy in myoblasts (Supplementary Figures 2C,D). These data, together with the results depicted in Figure 2, indicate that PC2 induces atrophy in C2C12 skeletal muscle cells by an autophagy-independent mechanism.

### PC2 Modulates mTOR Pathway in C2C12 Myotubes

**Figure 4:**
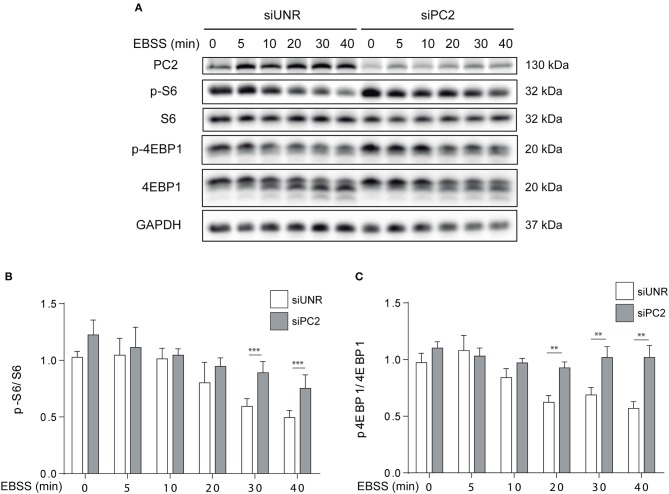
PC2 modulates the mTOR pathway. PC2 was downregulated in C2C12 myotubes using a specific siRNA against PC2 (siPC2) and an unrelated siRNA (siUNR) was used as control **(A–C)**. C2C12 myotubes downregulated for PC2 were submitted to starvation with EBSS for 0, 5, 10, 20, 30, and 40 min **(A–C)**. Then, whole lysates were resolved by western blot and Polycystin-2 (PC2), S6, P-S6 Ser235/236, 4EBP1, and P-4EBP1 Thr37/46 levels were evaluated. GAPDH was used as loading control. Representative gels are showed in **(A)** and relative levels of P-S6/S6 and P-4EBP1/4EBP1 are depicted in the graphs in **(B,C)** (mean ± S.E.M., *n* = 3, **p < 0.01, ***p < 0.001).

It is known that the mTOR and AKT axis not only regulates hypertrophy, but also atrophy in skeletal muscle *in vivo* and *in vitro* (11, 15, 23, 24). Given that it is well-known that starvation affects mTOR (14) and that our results indicate PC2 is required for starvation-induced atrophy, we evaluated if PC2 modulates mTOR signaling in C2C12 myotubes. To this aim, myotubes were submitted to siRNA-mediated down regulation of PC2 or transfected with an unrelated siRNA, siUNR. Then, cells were subjected to starvation with EBSS medium at different time points. Our results show that down regulation of PC2 prevents the decrease in the phosphorylation of the downstream-mTOR proteins, S6, and 4EBP1, when atrophy is induced by starvation (Figures 4A–C). Another signaling pathway implicated in the induction of atrophy is the inactivation of AKT with the subsequent translocation from the cytoplasm to the nucleus of the forkhead box O (FoXO) proteins (25, 26). In this regard, our data showed that downregulation of PC2 does not regulate starvation-induced dephosphorylation of AKT on Ser473 (Supplementary Figures 3A,B). Altogether, these results suggest that PC2 regulates starvation-induced atrophy via mTORC1 in C2C12 myotubes, independently of AKT (Figure 4A–C and Supplementary Figures 3A,B).

## Discussion

Severe and progressive muscle atrophy is observed in different human pathological conditions and several of the molecular mechanism that control atrophy remains elusive (1–4). Previous studies of our group demonstrated that PC2 positively regulates autophagy and that depending on the stimuli, cell type and tissue the modulation of mTOR is implicated in the process (16, 17). Furthermore, downregulation of PC2 inhibits autophagy induced by nutrient deprivation, rapamycin, and hypertonicity in different types of cells, while its overexpression triggers autophagy (16, 17). The requirement of PC2 for autophagy induction has also been demonstrated *in vivo*, in mouse heart tissues, where starvation-induced autophagy was inhibited in the heart mouse knocked-out for PC2 in cardiomyocytes (17). Given that massive autophagy has been observed in different models of atrophy and that there are no evidences about the role of PC2 in skeletal muscle cell models, we evaluated here if PC2 is regulating atrophy in C2C12 myotubes induced by classical stimuli of autophagy, such as nutrient deprivation or treatment with rapamycin. We observed that starvation and rapamycin induced atrophy in C2C12 myotubes and that PC2 is necessary in this process (Figures 1, 2). Although, other studies have showed that PC2 has a role in the control of autophagy, specifically in human embryonic stem cell-derived cardiomyocytes, neonatal cardiomyocytes, and renal epithelial cells (16, 18, 19) we did not observe that PC2 regulates autophagy in C2C12 myotubes, as assessed by the conversion of LC3 I to LC3 II. Importantly, previous studies that identified a role for PC2 in the regulation of autophagy in renal epithelial cells showed that it is related with pathways activated by fluid flow-induced mechanical stress, where the role of primary cilium has been shown to be key. In this regard, we can speculate that the intracellular location of PC2 as well as the type of stimuli or condition that affects autophagy might differentially affect PC2 function and/or autophagy. Here, our data showed that modulation of atrophy by PC2 is autophagy-independent given that PC2 did not modulate rapamycin-induced LC3 I to LC3 II conversion (Supplementary Figure 1) and consistently, autophagy was not altered following PC2 over expression-induced atrophy conditions (Figure 3 and Supplementary Figure 2).

mTOR has a pro-myogenic role given that it positively regulates protein synthesis (14). Indeed, skeletal muscle-specific ablation of Raptor, an adapter protein of the mTORC1, or deletion of the mTORC1 substrate S6 kinase, causes muscle dystrophy, and suppresses muscle growth adaptations to nutrient availability, respectively (27). Given the pro-myogenic relevance of mTOR, we evaluated if PC2 regulates atrophy by modulation of the mTOR pathway. Our results showed that downregulation of PC2 inhibited starvation-induced dephosphorylation of the protein S6, a key downstream target of mTOR, suggesting that regulation of atrophy by PC2 can be mediated by the modulation of mTOR signaling (Figure 4). Studies have shown that the role of mTOR not only has been related with the modulation of the size of the muscle fibers but its function has been also observed in other types of cells. Indeed, MDCK cells show a dramatically increase in cell size when the mTOR pathway is activated (28). Interestingly, our results showed that although the down regulation of PC2 attenuates the effects of starvation on the mTOR pathway, however, despite this it was unable to inhibit autophagy induced by rapamycin (Supplementary Figure 1), indicating that PC2, by modulating mTOR, and not autophagy, regulates atrophy in C2C12 myotubes.

To elucidate the mechanism by which PC2 regulates atrophy, we evaluated the phosphorylation status of AKT, which controls the activation of the transcription factor FoXO1, a known regulator of atrophy-related genes expression. Even if the status of phosphorylation of AKT was sensitive to starvation, it was unaffected by PC2 downregulation, indicating that AKT pathway does not participate in the regulation of atrophy by PC2. In this regard, other studies have also shown events of atrophy independent of AKT/FOXO1 pathway, especially in glucocorticoid-induced muscle atrophy (29).

Regarding the function of PC2, it is a Ca^2+^-permeable ion channels with a relevant role in the maintenance of the cytosolic Ca^2+^ (30). Indeed, mutations in PC2 lead to impaired calcium homeostasis in cardiac muscle which predispose cardiomyopathies *in vivo* (31). Thus, given that PC2 was required for starvation- and rapamycin-induced atrophy and that autophagy was not implicated in the mechanism by which PC2 regulates atrophy, it is possible that calcium microdomains controlled by PC2 may be also required to induce atrophy in C2C12. In fact, agents such as angiotensin II, tumor necrosis factor-α (TNFα) and lipopolysaccharide not only induce muscle atrophy but also a rise in Ca^2+^ which is necessary for both proteolysis and decreasing in protein synthesis in muscle cells (29, 32–34). This work did not evaluate the role of Ca^2+^. However, it could be addressed in future studies.

In conclusion, all these findings reveal a novel role of PC2 on the regulation of atrophy, which is mediated by the modulation of mTOR in C2C12 myotubes.

## Materials and Methods

### Cell Culture and Treatments

C2C12 myoblasts were purchased in Sigma-Aldrich (Cat N° 91031101-1VL). Cells were grown in DMEM (glucose 4.5 g/L) containing L-glutamine, 110 mg/L sodium pyruvate, 10% FBS, and 10 mM HEPES. Differentiation of C2C12 myoblast to myotubes was performed by culturing cells in DMEM (glucose 4.5 g/L) containing L-glutamine, 110mg/L sodium pyruvate, 10mM HEPES and 2% horse serum by 1 week. Media, supplements and reagents for cell culture were purchased from Gibco-Invitrogen (Carlsbad, USA). Cells were submitted to nutrient deprivation by culture of cells in Earle's Balanced Salt Solution medium, EBSS (Sigma-Aldrich, St. Louis, USA). Rapamycin and Bafilomycin A1 were purchased in Sigma-Aldrich. All experiments were independently repeated at least three times.

### siRNA Transfection and Adenovirus Infection

siRNAs were purchased by Sigma-Aldrich Corporation. An unrelated-siRNA sequence was used as negative control. Lipofectamine iMax (Invitrogen) and Optimem culture medium were used for siRNA transfections. Thirty-six hours after transfection cells were stimulated. Protein quantification of the targeted protein was used to evaluate the efficiency of the different siRNAs. For adenovirus-mediated protein overexpression, cells were incubated for 12h with the AdPC2 adenovirus.

### Western Blot Analysis

Protein samples of C2C12 myoblasts cells were prepared in M-PER lysis buffer (Thermo Scientific) supplemented with protease and phosphatase inhibitors (ROCHE). Aliquots of the extracted proteins (~30 μg/lane) were resolved in 10 or 12% SDS-PAGE gels and then subjected to immunoblotting using antibodies specific for Myogenin (mouse monoclonal IgG clone F5D, cat. n° sc-12732; Santa Cruz Biotechnology), Caveolin-3 (Mouse monoclonal IgG clone 26, cat. n° 610420; BD Bioscience Laboratories^TM^), GAPDH (mouse monoclonal IgG, cat n° MAB274; Chemicon International), 4EBP1(rabbit polyclonal IgG cat n° 9452; Cell Signaling Technology), P-4EBP1 Thr37/46 (rabbit polyclonal IgG clone 236B4, cat n° 2855; Cell Signaling Technology), LC3 I and II (rabbit polyclonal IgG, cat n° 9748; Cell Signaling Technology), p62/SQSTM1 (rabbit polyclonal IgG, cat n°, NBP1-42822; Novus Biologicals), PC2 (rabbit polyclonal IgG clone H-280, cat. n° sc-25749; Santa Cruz Biotechnology), AKT/PKB (mouse monoclonal IgG cat n°2966; Cell Signaling Technology), P-AKT/PKB Ser473 (rabbit polyclonal IgG cat n°4060; Cell Signaling Technology), S6 (mouse monoclonal IgG clone 54D2, cat n° 2317; Cell Signaling Technology), P-S6 Ser235/236 (rabbit polyclonal IgG clone 236B4, cat n° 2211; Cell Signaling Technology). Then, membranes were incubated with secondary goat anti-mouse or anti-rabbit IgG conjugated to horseradish peroxidase (SouthernBiotech, Birmingham, USA) prior to revelation by means of ECL Detection Kit (Amersham Pharmacia, Pittsburgh, USA). Gels were analyzed and quantified with the software ImageJ (http://rsb.info.nih.gov/ij/).

### Confocal and Fluorescence Microscopy

Following treatments cells were washed twice with ice-cold PBS, fixed in paraformaldehyde (4% w/v) for 15min, permeabilized with Triton 0.1%, PBS for 10 min and blocked in 3% BSA-PBS for 1h. Nuclei were counterstained with Hoechst 33342 (1 μg/mL) (Molecular Probes). Fluorescence and confocal fluorescence images were captured using an IRE2 microscope equipped with a DC300F camera (both from Leica Microsystems GmbH, Wetzlar, Germany) and an LSM 510 microscope (Carl Zeiss, Jena, Germany). Images were analyzed with the software ImageJ (http://rsb.info.nih.gov/ij/).

### Myotube Diameter Measurement

Myoblasts were differentiated into myotubes by culturing cells in DMEM containing 2% horse serum for 1 week. Then, myotubes were used to evaluate the cell diameter. Briefly, images were obtained with an epifluorescent microscope (Nikon Eclipse TI) and analyzed by a transverse line across the myotube. Myotubes with more than three nuclei were used for diameter measurements. We draw the line of distance across the myotube, which represents the myotube diameter (μm). At least three diameters per myotube were measured and at least 100 myotubes per well were analyzed using ImageJ Software. Data is presented as fold changes relative to control levels.

## Results and statistical analysis

Results are shown as mean ± S.E.M. from at least three independent experiments. Statistical analyses were performed using Student's *t*–test when analyzing two independent groups, one-way ANOVA for more than two independent groups and two-way ANOVA for two independent variables followed by a Sidak *post-hoc* test (GraphPad Software Inc.). *P* < 0.05 was considered to be statistically significant.

## Author Contributions

CK, DP-O, CH, NH-M, and AM-B performed the experiments. MH-C performed experiments and statistical analysis in the second round of revisions. SL and MB contributed to the experimental design and manuscript preparation. EM contributed to the experimental design and image analysis. VP performed mitochondrial morphology studies in the second round of revisions. RT and AC conceived the project and contributed to manuscript preparation.

### Conflict of Interest Statement

The authors declare that the research was conducted in the absence of any commercial or financial relationships that could be construed as a potential conflict of interest.

## Supplementary Material

Supplementary Figure 1 PC2 is not required for rapamycin-induced autophagy. PC2 was downregulated in C2C12 myotubes using a specific siRNA against PC2 (siPC2) **(A,B)**. Unrelated siRNA (siUNR) was used as control. C2C12 myotubes downregulated for PC2 were treated with rapamycin 0.1 μM **(A–B)** for 0 and 6h. Whole lysates were resolved by western blot and Polycystin-2 (PC2), LC3 I and LC3II were evaluated. GAPDH was used as loading control. Representative gel is showed in **(A)** and the relative levels of LC3 II/LC3 I are depicted in the graph in **(B)** (mean ± S.E.M., *n* = 3, ^**^*p* < 0.01, ^***^*p* < 0.001).

Supplementary Figure 2 Overexpression of PC2 does not regulate autophagy both in C2C12 myotubes and myoblasts. PC2 was overexpressed in C2C12 myotubes **(A,B)** or myoblast **(C,D)** for 24h by using an adenovirus for PC2 (Ad PC2). Empty adenovirus was used as control (Ad Co). Whole lysates were resolved by western blot and Polycystin-2 (PC2), LC3 I, and LC3II were evaluated. GAPDH was used as loading control. Representative gels for myotubes and myoblasts are showed in **(A)** and **(C)**, respectively. Relative levels of LC3 I to LC3 II turnover in myotubes and myoblasts are depicted in the graph in **(C)** and **(D)**, respectively (mean ± S.E.M., *n* = 3).

Supplementary Figure 3 PC2 does not regulate AKT/PBK pathway. PC2 was downregulated in C2C12 myotubes by transfection with a specific siRNA against PC2 (siPC2). Unrelated siRNA (siUNR) was used as control. Then, cultures were submitted to starvation with EBSS **(A,B)** for 0, 5, 10, 20, 30, and 40 min. C2C12 myotubes whole lysates were resolved by western blot and total AKT/PKB and P-AKT/PKB Ser473 levels were evaluated by the use of specific antibodies. GAPDH was used as loading control. Representative gels are showed in **(A)** and relative levels of P-AKT/AKT are depicted in the graphs in **(B)** (mean ± S.E.M., *n* = 3).

## Abbreviations

AU: arbitrary units
Baf A1: Bafilomycin A1
mTOR: mechanistic target of rapamycin
PC2: polycystin-2
PKD: polycystic kidney disease
VPS34: vacuolar protein sorting 34
4EBP1: 4E-binding protein 1
ULK1: 51-like kinase 1 protein
Cav3: Caveolin-3
Myo: Myogenin
LC3: light chain.
