# Supplementary figures and images for: Polycystin-2 Is Required for Starvation- and Rapamycin-Induced Atrophy in Myotubes
Source: Front Endocrinol (Lausanne). 2019 May 8;10:280. doi: 10.3389/fendo.2019.00280 (PMC6517509; doi:10.3389/fendo.2019.00280)

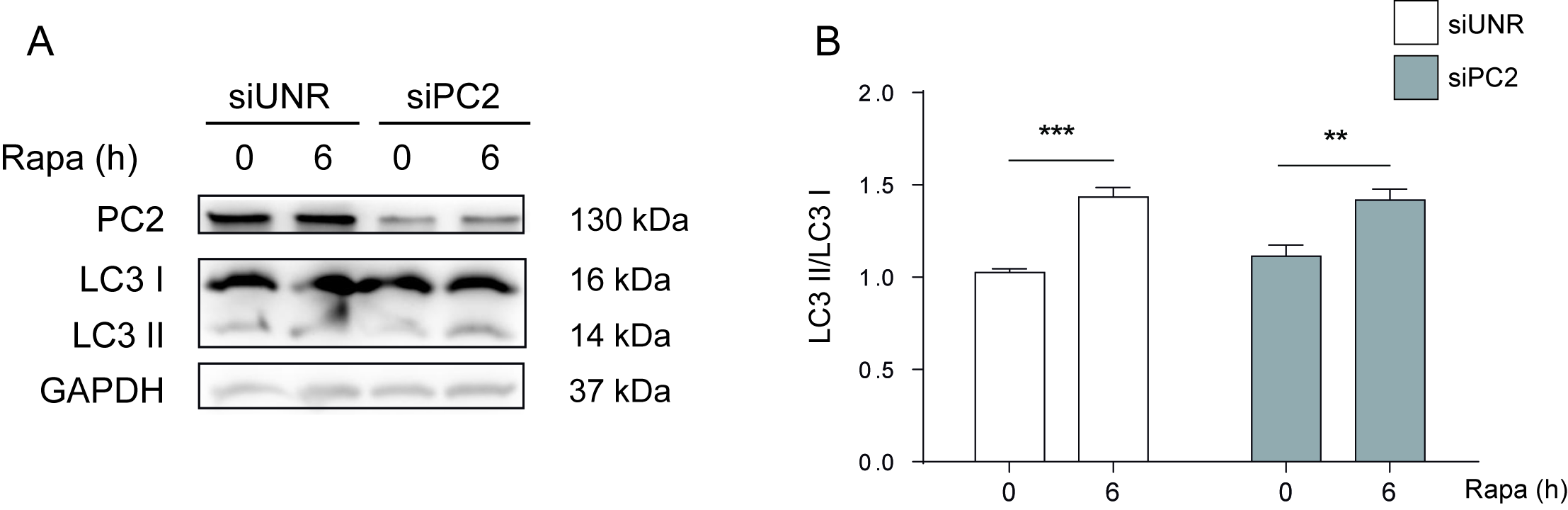

Supplement: Supplementary Figure 1 — PC2 is not required for rapamycin-induced autophagy. PC2 was downregulated in C2C12 myotubes using a specific siRNA against PC2 (siPC2) (A,B). Unrelated siRNA (siUNR) was used as control. C2C12 myotubes downregulated for PC2 were treated with rapamycin 0.1 μM (A–B) for 0 and 6h. Whole lysates were resolved by western blot and Polycystin-2 (PC2), LC3 I and LC3II were evaluated. GAPDH was used as loading control. Representative gel is showed in (A) and the relative levels of LC3 II/LC3 I are depicted in the graph in (B) (mean ± S.E.M., n = 3, **p < 0.01, ***p < 0.001). [file Image_1.TIF]

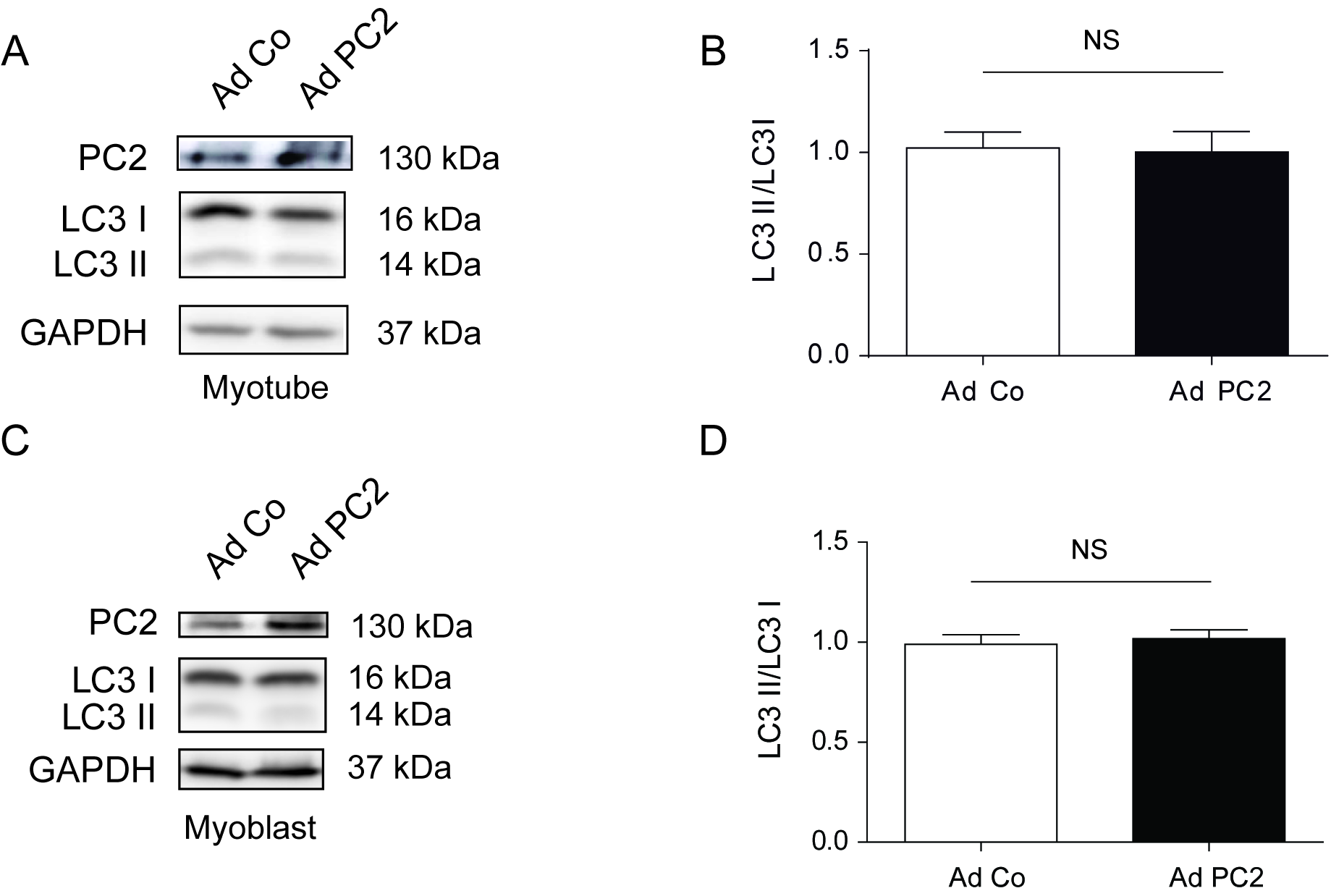

Supplement: Supplementary Figure 2 — Overexpression of PC2 does not regulate autophagy both in C2C12 myotubes and myoblasts. PC2 was overexpressed in C2C12 myotubes (A,B) or myoblast (C,D) for 24h by using an adenovirus for PC2 (Ad PC2). Empty adenovirus was used as control (Ad Co). Whole lysates were resolved by western blot and Polycystin-2 (PC2), LC3 I, and LC3II were evaluated. GAPDH was used as loading control. Representative gels for myotubes and myoblasts are showed in (A) and (C), respectively. Relative levels of LC3 I to LC3 II turnover in myotubes and myoblasts are depicted in the graph in (C) and (D), respectively (mean ± S.E.M., n = 3). [file Image_2.TIF]

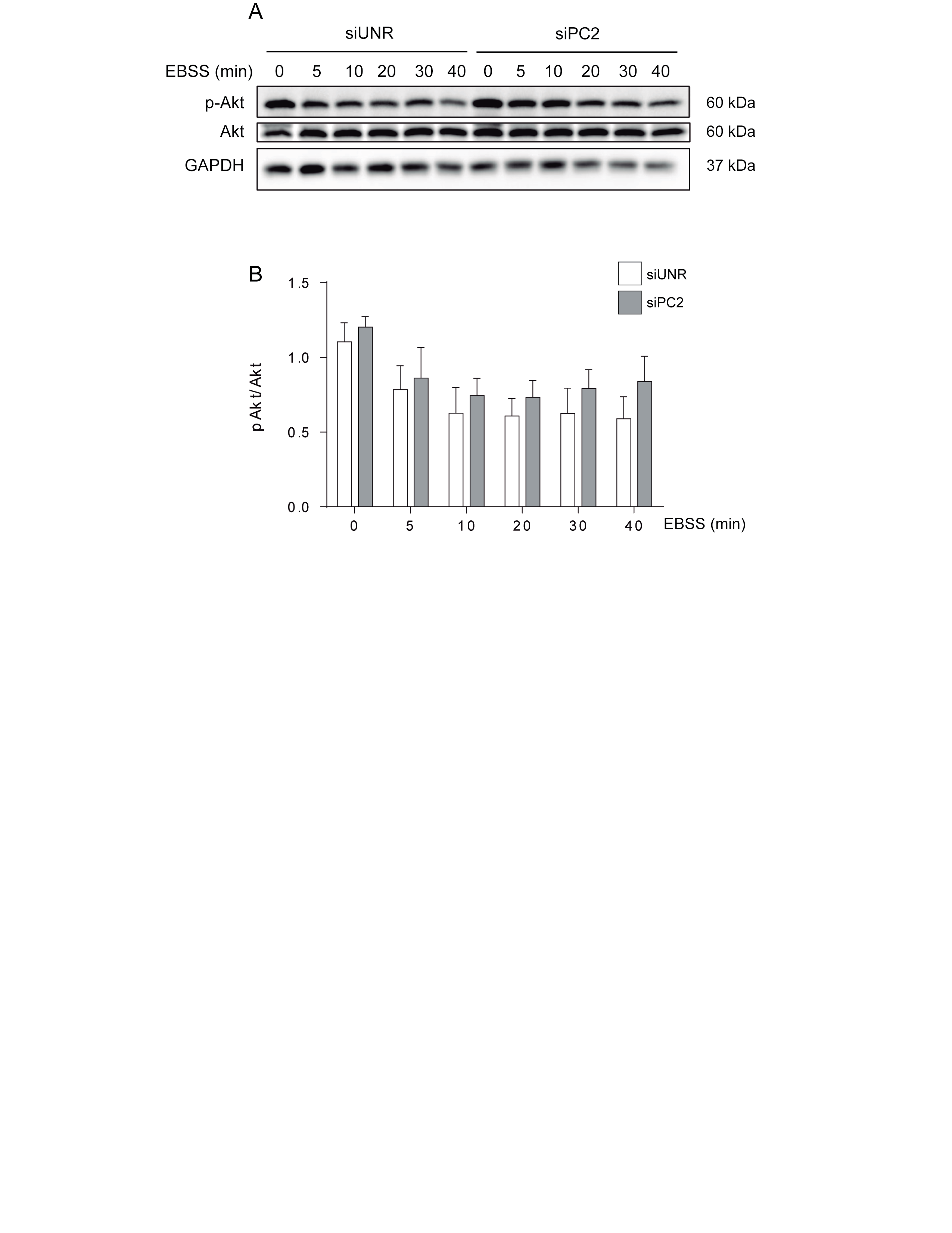

Supplement: Supplementary Figure 3 — PC2 does not regulate AKT/PBK pathway. PC2 was downregulated in C2C12 myotubes by transfection with a specific siRNA against PC2 (siPC2). Unrelated siRNA (siUNR) was used as control. Then, cultures were submitted to starvation with EBSS (A,B) for 0, 5, 10, 20, 30, and 40 min. C2C12 myotubes whole lysates were resolved by western blot and total AKT/PKB and P-AKT/PKB Ser473 levels were evaluated by the use of specific antibodies. GAPDH was used as loading control. Representative gels are showed in (A) and relative levels of P-AKT/AKT are depicted in the graphs in (B) (mean ± S.E.M., n = 3). [file Image_3.TIF]
